# Outcomes following B3/B4 needle core biopsy in South East London Breast Screening Service 2000 to 2010

**DOI:** 10.1186/bcr2954

**Published:** 2011-11-04

**Authors:** K Lynes, I Nikolopoulos, N Akbar, M Michell, K Thakur

**Affiliations:** 1 Queen Elizabeth Hospital, South London Healthcare NHS Trust, London, UK; 2 King's College Hospital NHS Foundation Trust, London, UK; 3 South East London Cancer Network, London, UK

## Introduction

Needle core biopsy (NCB) is frequently used in assessing screen-detected breast lesions. Uncertainty remains over appropriate management of NCBs reported as 'uncertain malignant potential' (B3) or 'suspicious of malignancy' (B4). This study aims to analyse a large screening dataset to establish positive predictive values (PPVs) for malignancy on excision biopsy, for different classifications of B3 and for B4 NCBs.

## Methods

Retrospective analysis was conducted of prospectively collected data for South East London Breast Screening Service. A total of 5,324 patients underwent NCB between 2000 and 2010, including 14G (ultrasound-guided) and 10G vacuum-assisted biopsies (stereo-guided). A total of 444 were B3 (8.3%) and 38 (0.7%) were B4. NCBs reported as B3 were classified by pathological subtype and PPVs for malignancy calculated for each subtype. Outcomes following B4 NCB were also assessed.

## Results

The overall PPV for malignancy for B3 NCBs was 25% and for B4 NCBs was 74%. The PPVs for each subtype of B3 classification were as follows: papillary 14%, atypical intraductal epithelial proliferation 35%, phyllodes 11%, lobular 47%, complex sclerosing lesion/radial scar 6%, columnar cell 29%.

## Conclusion

Our findings indicate that NCBs reported as B4 should be excised as they have a high likelihood of malignancy on excision biopsy. The PPVs for subtypes of B3 NCBs vary considerably. However, B3 subtypes with atypia should be treated with a higher level of suspicion and preferably be surgically excised. Decisions regarding further assessment should be made in a multidisciplinary setting.

